# Progress Confirmed for *Pharmaceuticals* in 2016

**DOI:** 10.3390/ph10010001

**Published:** 2016-12-26

**Authors:** Jean Jacques Vanden Eynde

**Affiliations:** Editor-in-Chief, MDPI AG, St. Alban-Anlage 66, Basel CH-4052, Switzerland; jean-jacques.vandeneynde@ex.umons.ac.be

## Looking Back on 2016

As explained in the editorial written 12 months ago [1], 2015 was considered as a transition year for our journal. We are proud to let you know that, thanks to your continuous support, *Pharmaceuticals* improved its ranking [2] in the field of molecular medicine, shifting in 2015 from the third quartile to the second quartile. For the third year in a row, *Pharmaceuticals* confirms its position in the first quartile in the category of pharmaceutical science. Moreover, papers published in our journal in 2013 and 2014 have been cited 4.5 times, on average, in 2015.

It is my pleasure to confirm the progress recorded in 2016. Indeed, the number of published manuscripts has jumped from 45 to 79 in 2016 volume. This includes submissions received in 10 completed Special Issues. They were dedicated to the First International Electronic Conference on Medicinal Chemistry [3] (Guest Editor: J.J. Vanden Eynde), Antimicrobial Peptides [4] (Guest Editor: G. Wang), Transient Receptor Potential Channels [5] (Guest Editors: A. Szallasi and S.M. Huang), the 100th Anniversary of the Discovery of Heparin [6] (Guest Editors: M.M.M. Pinto, M.E. de Sousa, and M.C. da Silva), Nanobiotechnology in Medicinal Chemistry [7] (Guest Editors: A.M. Grumezescu and A.M. Holban), the Eleventh National Meeting of Organic Chemistry and the Fourth Meeting of Therapeutic Chemistry in Portugal [8] (Guest Editors: V. Freitas and M.E. de Sousa), New Challenges in Radiochemistry [9] (Guest Editor: M. Monclus), Aptamers [10] (Guest Editors: A. Berzal-Herranz and C. Romero-Lopez), New Drugs in Hematology [11] (Guest Editor: L.J. Costa), and the Eighth International Conference on Protein Kinase CK2 [12] (Guest Editors: J. Jose, M. Le Borgne, L.A. Pinna, M. Montenarh). In the meantime, the Special Issue entitled “Choices of the Journal”, launched in 2015, has now been converted into a topical collection [13].

In 2016, eight distinguished scientists joined the Editorial Board: Alfredo Berzal-Herranz (Instituto de Parasitología y Biomedicina López-Neyra, Granada, Spain), Thierry Besson (Université de Rouen, Mont Saint-Aignan, France), Francois Dufrasne (Université Libre de Bruxelles, Bruxelles, Belgium), Marc Le Borgne (Université Lyon 1, Lyon, France), Andrea Porcheddu (Università degli Studi di Cagliari, Monserrato, Italy), Osvaldo A. Santos-Filho (Universidade Federal do Rio de Janeiro, Rio de Janeiro, Brazil), Gary J. Stephens (University of Reading, Reading, UK), and Nigel Yarlett (Pace University, New York, NY, USA).

*Pharmaceuticals* confirmed its status as an international journal by being present as a media partner, an exhibitor, or a sponsor at national and international meetings among which were:

- Biosensors 2016, Twenty-sixth Anniversary World Congress on Biosensors, 25–27 May, Gothenburg, Sweden;
- Thirtieth France-Belgium Meeting of Pharmacochemistry, 25–27 May, Amboise, France;
- Aptamers in Bordeaux 2016, 24–25 June, France;
- CLINAM 9/2016 Conference and Exhibition, European and Global Summit for Cutting-Edge Medicine: Clinical Nanomedicine and Targeted Medicine, Enabling Technologies for Personalized Medicine, 26–29 June, Basel, Switzerland;
- ACS National Meeting and Exhibition, 21–23 August, Philadelphia, PA, USA;
- Réunion du Groupement des Pharmacochimistes de l’Arc Atlantique, 25–26 August, Angers, France;
- Eighth International Conference on Protein Kinase CK2, 6–9 September, Homburg, Germany;
- Thirteenth European Biological Inorganic Chemistry (EuroBIC 13) Conference, 28 August–1 September, Budapest, Hungary;
- Twenty-fourth National Meeting in Medicinal Chemistry, 11–14 September, Perugia, Italy.
- Fourth Conference on Innovation in Drug Delivery: Site-Specific Drug Delivery, 25–28 September, Antibes-Juan-les-Pins, France;

*Pharmaceuticals* was the organizer of the Second International Electronic Conference on Medicinal Chemistry [14]. The event gathered more than 150 authors from 22 countries (Austria, Belgium, Brazil, France, Georgia, Germany, India, Ireland, Malta, Pakistan, Portugal, Romania, Russia, Serbia, Slovakia, Spain, Switzerland, Taiwan, Turkey, Ukraine, USA, and Venezuela). It attracted 14 media partners (Analis, Bionity.com, Chemeurope.com, Cherry Biotech, Elveflow, Eurisotop, Hielscher, Latoxan, Magritek, MDPI AG, Reaction Biology Corp, Sairem, SnapDragon, and Wylton) who had the opportunity to inaugurate our new virtual exhibition hall. The best presentation, as elected by the Scientific Committee of the Conference, was the work entitled “Design, Synthesis and Biological Activity of Furoxan Derivatives against Multi-drug Resistant Tuberculosis” [15], submitted by G. Fernandes, P. Souza, C.M. Chin, F. Pavan, and J.L. Dos Santos from the University Estadual Paulista at Araraquara, Brazil. It has been rewarded by a special travel grant offered by our journal.

Mentions should be made, in addition, that we received 83 outstanding applications for the 2016 *Pharmaceuticals* Travel Award for Young Investigators [16]. After a tied selection, the grant has been awarded to Dr. Zesergio Melo Jerez, of Colombia, who obtained his PhD degree in 2014 from the Universidad Nacional Autónoma de México, México City, México. The award helped him to present the oral communication entitled “Vasoinhibins Block NGF-Induced Neurite Outgrowth and Decrease Survival of PC12 Cells” at the Thirty-ninth Annual Meeting of the Japan Neuroscience Society (20–22 July, Yokohama, Japan).

## Looking Forward to 2017

In 2017, we shall pursue our efforts to raise the journal to a better position still and to increase its visibility. Therefore, we can already announce that *Pharmaceuticals* will offer a travel grant to help support a young investigator in her/his professional career. Details of the competition can be found on our website [17]. The journal will be present at the National Meeting and Exposition of the American Chemical Society in San Francisco, USA (2–6 April, 2017) and other conferences worldwide. It will also launch the Third International Electronic Conference on Medicinal Chemistry which will take place in November on http://sciforum.net/conference/ecmc-3.

It is my pleasure to end this editorial by wishing you a healthy and prosperous new year. This is also the opportunity for me to warmly thank, for their confidence, our authors, readers, and reviewers, as well as the scientists of the editorial board, members of our teams in Basel, Barcelona, Beijing, and Wuhan, and our sponsors.
